# Sequence diversity and evolution of a group of iflaviruses associated with ticks

**DOI:** 10.1007/s00705-021-05060-8

**Published:** 2021-04-19

**Authors:** Romain Daveu, Caroline Hervet, Louane Sigrist, Davide Sassera, Aaron Jex, Karine Labadie, Jean-Marc Aury, Olivier Plantard, Claude Rispe

**Affiliations:** 1 INRAE, Oniris, BIOEPAR, Nantes, France; 2 Department of Biology and Biotechnology “L. Spallanzani”, University of Pavia, Pavia, Italy; 3 Population Health and Immunity Division, Walter and Eliza Hall Institute of Medical Research, Parkville, VIC 3052 Australia; 4 Faculty of Veterinary and Agricultural Sciences, The University of Melbourne, Parkville, VIC 3010 Australia; 5 Genoscope, Institut de biologie François-Jacob, Commissariat à l’Energie Atomique (CEA), Université Paris-Saclay, Evry, France; 6 Génomique Métabolique, Genoscope, Institut François Jacob, CEA, CNRS, Univ Evry, Université Paris-Saclay, 91057 Evry, France

## Abstract

We studied a group of tick-associated viruses with characteristics of members of the family *Iflaviridae*, a family of viruses frequently found in arthropods. Our aim was to gain insight into the evolutionary dynamics of this group of viruses, which may be linked to the biology of ticks. We explored assembled RNA-Seq data sets for different species of ticks. We identified members of five different iflavirus species, four of them novel, and discovered nine new genome sequences, including variants. Five variants represented a virus species associated with *Ixodes ricinus*. Unexpectedly, a sequence found in the *Ixodes scapularis* cell line ISE6 was nearly identical to the sequences of *I. ricinus* variants, suggesting a contamination of this cell line by *I. ricinus* material. Analysing patterns of substitutions between these variants, we detected a strong excess of synonymous mutations, suggesting evolution under strong positive selection. The phylogenies of the viruses and of their tick hosts were not congruent, suggesting recurrent host changes across tick genera during their evolution. Overall, our work constitutes a step in the understanding of the interactions between this family of viruses and ticks.

## Introduction

Ticks are blood-feeding parasites, which makes of them a "hub" for a number of microorganisms (bacterial pathogens and symbionts, viruses, protozoa) that potentially interact with ticks and their vertebrate hosts. The discovery of new RNA viruses has rapidly increased in recent years, and they appear to be far more prevalent and diverse than previously expected, especially in invertebrates [1, 2]. High-throughput transcriptome sequencing (or RNA-Seq) has proven to be the tool of choice for characterizing the genomes of these viruses in many types of arthropods [2, 3], including ticks [4, 5], as this technique does not require known sequences to be targeted, while allowing the retrieval of contigs that may contain full viral genome sequences, given a sufficient coverage and sufficient prevalence of the virus in the analysed samples. The *Iflaviridae* are a family of RNA viruses within the order *Picornavirales.* They are non-enveloped and have a positive-sense, single-stranded non-segmented genome of ~9-11 kb [6]. This family of viruses has been shown to have a privileged association with insects, being detected in a growing number of species belonging to several orders. Iflavirus infections can range from asymptomatic to severe, causing developmental anomalies (e.g., deformed wing virus in honey bees) or death [6]. Iflaviruses have also been detected in parasitic mites (subphylum Chelicerata) [7]. Recently, genomes of viruses belonging to the family *Iflaviridae* have been detected in pools of tick species [2], in an endemic Australian tick species (Ixodes holocyclus iflavirus [IhIV]) [8], in a tick species associated with marine birds, *Ixodes uriae* [9, 10] (Gerbovitch virus), and in the ISE6 tick cell line (Ixodes scapularis iflavirus [ISIV]) [11]. Overall, members of the family *Iflaviridae* are among the most common viruses that have been identified in different species of ticks, although many aspects of their biology are still unknown, including their mode of transmission, their effect on the fitness of the tick host, and the phylogenetic relationships among members of this family. For this reason, we decided to explore the many transcriptome data sets that have been published for different tick species, searching for new sequences of members of the family *Iflaviridae*. We identified nine new genome sequences representing five different species of tick-associated iflaviruses, including several variants of the same virus species in *Ixodes ricinus*, and studied the evolution and phylogenetic relationships of this group.

## Materials and methods

### Search for iflavirus sequences

We searched for sequences matching iflaviruses in public tick transcriptome assemblies (available from TSA, GenBank, as of August 2020). For each transcriptome assembly, we searched for matches using tblastn [12], using the polyprotein sequence of ISIV (accession number BBD75427) as a query and RNA-Seq contigs (nucleotide sequences) as targets. Of note, several of these transcriptomes were produced by our research group for a phylogenetic study of hard ticks [13] or for expression profiling studies of *I. ricinus* (BioProject accession numbers PRJEB40724 and PRJNA662253). We used low-stringency matching criteria (e-value threshold 1-e3) to ensure that even relatively distantly related sequences could be found. Since new genome sequences identified in our study were retrieved from transcriptome data, they represent uncultivated virus genomes (UViGs). Genomes were defined as representing novel species if their sequence identity to members of existing species was less than 90%, according to the species delineation criteria of the International Committee on Taxonomy of Viruses (ICTV) for iflaviruses (https://talk.ictvonline.org/ictv-reports/ictv_online_report/positive-sense-rna-viruses/w/iflaviridae). We also performed a BLASTp search on the nr database, selecting all of the matches described as being associated with ticks and adding them to our data set. This approach ensured that our study included all of the tick-associated iflavirus genome sequences published to date (as of September 2020).

### Phylogeny methods

Based on a recent phylogenetic study of iflaviruses [14], we included two outgroups, one associated with the mite *Tetranychus truncatus* (representing the closest outgroup to tick-associated iflaviruses) and the other associated with *Apis mellifera* (deformed wing virus [DWV]). We aligned amino acid sequences of all iflavirus polyprotein sequences using MUSCLE in MEGA X [15]. This alignment was filtered using Gblocks [16] to exclude poorly aligned regions and gaps. The filtered alignment comprised 1,149 amino acid positions. A phylogenetic ML tree was constructed using IQ-TREE [17]. The best model of substitution was determined using Model Finder [18], and branch support was assessed using 1000 ultrafast bootstrap replicates [19]. A graphical representation of the consensus ML tree was made using ITOL [20].

### Test of congruence between host and virus phylogenies

To test the congruence between virus and host phylogenies, we used the cophylogeny testing tool Jane 4 [21]. The host phylogeny was derived from a previous transcriptome-based phylogenetic study of hard ticks, using a large number of nuclear markers [13]. Three iflavirus genomes, obtained from pools of different species of ticks, could not be included in this analysis because it was impossible to assign a host species to these iflaviruses. We also collapsed branches containing virus sequences that were nearly identical in the virus phylogeny, since they can be considered to represent the same species. This resulted not only in eliminating two iflavirus sequences associated with *I. holocyclus* but also another group of six sequences, five of which were found in *I. ricinus* and one of which was found in a cell line of *I. scapularis* (see “Results”). The name given to this group was IricIV-ISIV.

### Estimation of genetic distances and evolutionary rates

We estimated genetic distances and evolutionary rates within two ensembles of sequences, one including ISIV and *I. ricinus* variants and the other including two variants associated with *I. holocyclus*, that grouped closely in the phylogenetic analysis. We first estimated genetic distances (number of substitutions per site) at both the nucleotide level and the amino acid level, using the maximum composite likelihood model [22] and the Poisson correction model [23], respectively, with the complete deletion option (all gaps excluded). This analysis was performed in MEGA X [15]. We then determined the ratios of non-synonymous to synonymous substitutions (dN/dS) using Codeml [24] with the one-ratio model for the ISIV + *I. ricinus* group and the pairwise estimate of dN/dS for the two variants found in *I. holocyclus*.

### Virus presence across different populations and stages of Ixodes ricinus

The abundance of virus genomes was assessed by mapping RNA-Seq reads to the genome sequence of the iflavirus IricIV-1. Because a very low abundance of a virus can be caused by library cross-contamination (index hopping), a library was considered positive only if the read count per million was above one, following an approach used in a recent study [10].

## Results

### Iflavirus sequences identified

We found five variants of a novel iflavirus associated with *Ixodes ricinu*s (Tables 1 and 2). These sequences were associated with different tissues or stages of the tick life cycle (in the GIDG transcriptome assembly) and with ticks from different geographical regions. All viral genome sequences obtained from *I. ricinus* were highly similar to the ISIV sequence obtained from a cell line of *I. scapular*is (~98% amino acid identity, while higher variation was detected at the nucleotide level, as shown below). Although the ISIV sequence was found in the transcriptome of a cell line from *I. scapularis*, there was no match with this sequence in the three available *de novo* assemblies obtained for three independently sequenced transcriptomes of *I. scapularis* (two of which were obtained from large collections of wild ticks, with a total of ~200 males and females from three locations for the GGIX TSA assembly). Novel iflaviruses were also identified in *Ixodes frontalis* (IfronIV), *Ixodes vespertilioni*s (IvespIV), and *Hyalomma dromedarii* (HydromIV) (Table 2). For the first two of these viruses, the retrieved sequences were incomplete (5' partial), and the sequenced region of the ORF comprised 2,437, and 1,945 amino acids, representing ~81% and ~65% of the complete genome, respectively. In the case of HydromIV, the match with the ISIV sequence was in two frames, suggesting a frameshift. This frameshift could be authentic, especially if this sequence corresponds to an endogenous viral element (EVE), given that EVEs do not necessarily maintain open reading frames [25]. A detailed analysis of reads mapping to the region of the frameshift showed that it was located in a homopolymeric (poly-A) region and that the reads were polymorphic in that region, most containing a deletion of one A compared to the contig. The corrected sequence, based on the majority of reads, contained an intact ORF and no frameshift. We therefore corrected this contig, and all subsequent analysis of this sequence was based on the corrected sequence. Additionally, a 5' partial sequence was also found in a transcriptome assembly of *I. holocyclus* (2,039 amino acids, IhIV-2). This sequence was similar but not identical to that of the first iflavirus genome (IhIV) identified for that tick species. The new sequences were deposited in the GenBank database (accession numbers in Table 1) with metadata specifying the source of these viruses, the assembly methods used, and the quality of the sequence data (following the guidelines for the Minimum Information about an Uncultivated Virus Genome, or MIUVIG [26]).

**Table 1:** List of iflavirus sequences found in tick transcriptomes (with tblastn or blastp, using the ISIV polyprotein sequence as a query). Columns: Species, Tissues (HEM: haematocytes, MG: midgut, MT: Malpighian tubules, OV: ovaries, SG: salivary glands, SYN: synganglion, WB: whole bodies) followed by details on the stages or conditions between parentheses, when available, Location of the sampling (or source of the strains), Accessions: GenBank accessions, protein and nucleotide, and if available, related TSA or BioProject accession between parentheses, Publication (or authors of the sequences), Percent identity with ISIV -% id at the amino acid level of the first hsp (tblastn)- and query range of the match. Lines in bold correspond to the nine iflavirus genome sequences newly discovered in the present study. For HydromIV, the sequence used was a corrected contig sequence

Species	Tissues and conditions	Location	Accession numbers	Publication	Identity	Match range(s)	Virus name
*Amblyomma americanum*	WB (wild questing ticks)	NY and Connecticut, USA	ASU47553.1, KX774633.1	Tokarz et al. 2018	65.8%	1142-2838	Lone star tick dicistrovirus
*Haemaphysalis flava*	WB	Japan	BBK20270.1, LC483655.1	Kobayashi et al. 2020	45.6%	63-2937	HflFV
*Hyalomma asiaticum*	WB	China	APG77501.1, KX883729.1	Shi et al. 2016	48.4%	379-2990	Bole Hyalomma asiaticum
***Hyalomma dromedarii***	**SG, wild ticks**	**Tunisia**	**BK012003 (GFGI01)**	**Bensaoud et al. 2018**	**40.0%**	**1-1880 and 2000-2989**	**HydromIV**
***Ixodes frontalis***	**WB**	**Carquefou, France**	**QPI13027.1, MT008333 (PRJNA528282)**	**Charrier et al. 2019**	**41.1%**	**560-2989**	**IfronIV**
*Ixodes holocyclus*	SG,WB,MG	QLD and NSW, Australia	AQZ42314.1, KY020412.1 (GIBQ01)	O'Brien et al. 2018	62.2%	1-2989	IhIV
***Ixodes holocyclus***	**WB**	**NSW, Australia**	**QPI13026.1, MT008332 (PRJNA528282)**	**Charrier et al. 2019**	**65.4%**	**913-2989**	**IhIV-2**
***Ixodes ricinus***	**SYN (wild unfed ticks)**	**Chizé, France**	**QPI13029.1, MT008330.1 (PRJEB40724)**	**Rispe et al (unpub.)**	**97.7%**	**1-2991**	**IricIV-1**
***Ixodes ricinus***	**SG, OV, MT (feeding adult females)**	**Neuchâtel (Switzerland), lab strain**	**QPI13030.1, MT008331 (PRJNA662253)**	**Daveu et al (unpub.)**	**98.4%**	**1-2991**	**IricIV-2**
***Ixodes ricinus***	**WB (various stages)**	**Czech Republic**	**BK012002 (GIDG01012278)**	**Vechtova et al. (unpub.)**	**98.1%**	**1-2991**	**IricIV-3**
***Ixodes ricinus***	**WB (wild nymphs)**	**Switzerland**	**QPI13031.1, MT050463 (PRJNA662080)**	**Rispe et al (unpub.)**	**98.3%**	**6-2991**	**IricIV-4**
***Ixodes ricinus***	**WB (wild nymphs)**	**Nancy, France**	**QPI13032.1, MT050464 (PRJNA662080)**	**Rispe et al (unpub.)**	**98.2%**	**1-2991**	**IricIV-5**
*Ixodes scapularis*	Cell line ISE6	-	BBD75427.1, LC094426.1	Nakao et al. 2017	100.0%	1-2991	ISIV
*Ixodes uriae*	WB	Antartic peninsula	QIS88066.1, MT025175.1	Wille et al. 2020	42.0%	383-2991	Gerbovich virus
***Ixodes verspertilionis***	**WB**	**Maine-et-Loire, France**	**QPI13028, MT008334 (PRJNA528282)**	**Charrier et al. 2019**	**68.2%**	**1016-2989**	**IvespIV**
Pool of tick species	WB	China	YP_009336552.1, NC_032764.1	Shi et al. 2016	38.6%	381-2991	Ht-V1
Pool of tick species	WB	China	YP_009336542.1, NC_032758.1	Shi et al. 2016	44.3%	391-2991	Ht-V2
Pool of tick species	WB	China	YP_009336533.1, NC_032751.1	Shi et al. 2016	46.1%	63-2989	Ht-V3

**Table 2:**
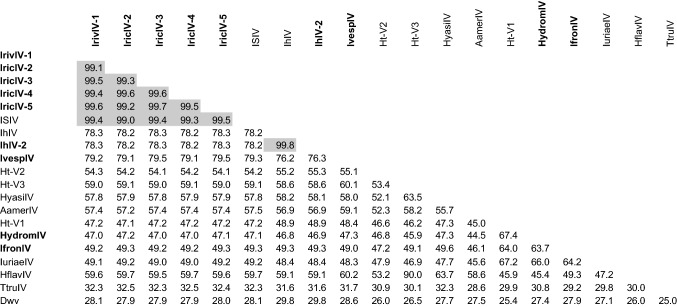
Percentage of amino acid identity between iflavirus polyprotein sequences, for each pair of sequences included in our study. In bold, sequences newly identified in this work. Values shaded in gray indicates identity above 90%, defining sequences that belong to the same species based on species delineation criteria of iflaviruses.

All other assemblies obtained from multiple genera of hard and soft ticks gave negative results. Finally, other sequences were found after a BLASTp search against the GenBank protein database (nr), which allowed the inclusion of sequences from viruses associated with *Amblyomma americanum* (lone star tick dicistrovirus), *Haemaphysalis flava* (HfFV)*, Hyalomma asiaticum* (Bole Hyalomma virus)*,* and *Ixodes holocyclus* (IhIV), *Ixodes uriae* (Gerbovich virus) as well as viruses from pools of tick species (Table 1).

### Genome organization

**Fig. 1:**
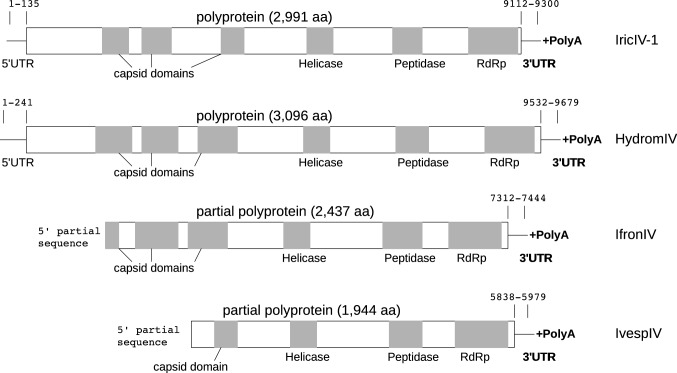
Genome organization of the four novel tick-associated iflaviruses discovered in this study, respectively found in *Ixodes ricinus* (IricIV-1), *Hyalomma dromedarii* (HydromIV), *Ixodes frontalis* (IfronIV), and *Ixodes vespertilionis* (IvespIV). ORFs were predicted and domains were searched with Interproscan [34]. Predicted domains are shown as grey bars.

We analyzed the genome organization of the four sequences we consider possible representatives of novel iflavirus species (Fig. 1). Two of them (for IricIV and HydromIV) appear to contain a complete ORF and both 5' and 3' UTRs. These sequences contain conserved domains, including three capsid domains, followed by a helicase, a peptidase, and an RNA-dependant RNA polymerase (RdRp) domain. Two other sequences (from IfronIV and IvespIV) were incomplete at the 5' end, lacking the 5' UTR and at least part of the capsid domains, while the other domains and the 3' UTRs were present.

### Phylogeny

**Fig. 2:**
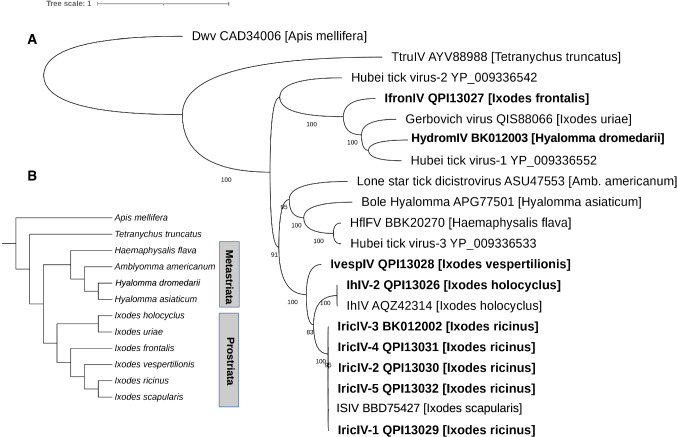
**A.** Maximum-likelihood phylogenetic tree of tick-associated iflaviruses. The tree was based on the amino acid sequence of iflavirus sequences found in the transcriptomes of different hard tick species (Acari; Parasitiformes; Ixodida; Ixodidae) and rooted with two outgroups, a honey bee iflavirus (deformed wing virus [DWV]) and an iflavirus associated with *Tetranychus truncatus* (Acari; Acariformes; Prostigmata), TtruIV. The host taxon is indicated in brackets. Details related to each taxon are given in Table 1. Taxon names in bold correspond to the nine iflavirus sequences discovered in the present study. Bootstrap support is indicated at the nodes. **B.** Expected topology of the phylogenetic tree of arthropod hosts of iflaviruses included in this study, based on reference 13 and on the delimitation of the two groups recognized within the family Ixodidae (Prostriata and Metastriata).

The iflavirus genome sequences included in our work are described in Table 1, including nine newly identified sequences from the present study (Fig. 2). The best-fit model (BIC criteria) was LG+G4. An ML tree (consensus from 1,000 bootstrap trees) showed that the five variants found in *I. ricinu*s and the ISIV sequence grouped closely together (see below for details on genetic distances). This group therefore represents variants of the same virus species. This was also the case of the two variants of *I. holocyclus.* Another iflavirus sequence, found in *I. vespertilionis*, formed a sister group with the two groups above. Two other iflavirus genomes, found in *I. frontalis* and *H. dromedarii*, respectively, grouped with Ht-V1 [2]. The latter sequence was obtained from a pool of tick species that contained members of several species of the Metastriata (non-*Ixodes* hard ticks) or soft tick species (Argasidae), but no members of the genus *Ixodes* [2]. Of note, a sequence found in *A. americanum* in a study by Tokarz et al. [5] corresponds to an iflavirus, although it was named "dicistrovirus" in that publication.

### Test of cophylogeny

**Fig. 3:**
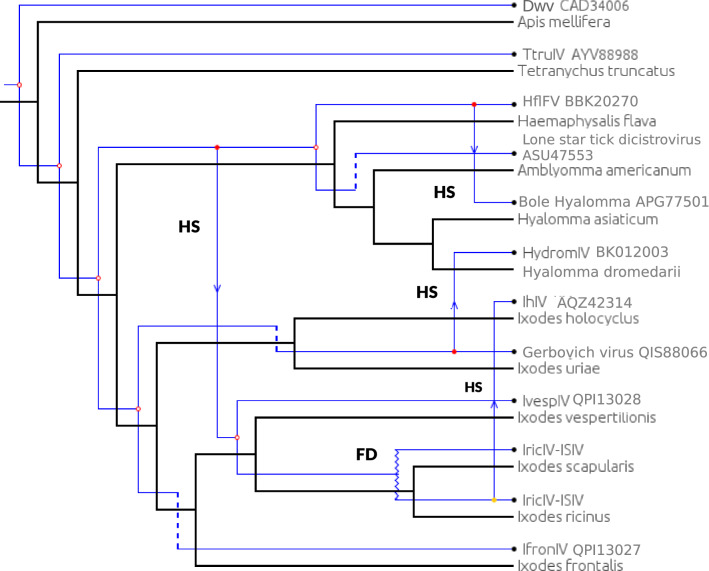
Test of the congruence between the phylogenies of tick-associated iflaviruses and of tick hosts, using Jane 4. This tree contains only part of the sequences analysed in Fig. 2 (three virus genomes found in pools of tick species could not be included). Iric-ISIV represents iflavirus sequences found either in *I. ricinus* or in a cell line of *I. scapularis* (these sequences being nearly identical). Open circles at the nodes indicate cophylogeny, HS and solid colored circles, red or yellow, indicate a host shift, FD indicates a failure to diverge

A test of cophylogeny was performed using Jane 4, allowing us to depict several possible coevolutionary scenarios, all of which had the same scoring and differed very little in structure (we present one of them in Fig. 3). For all scenarios, there was an initial event of cophylogeny due to the fact that all iflavirus genomes associated with ticks form a monophyletic clade, but after this conserved ancestral node, all scenarios included four instances of host switching. An additional anomaly was a "failure to diverge" between viruses associated with *I. ricinus* and *I. scapularis* (see “Discussion”).

### Distances among iflavirus genome sequences and rates

For the group comprising variants found in *I. ricinus* and the ISIV sequence, the estimated pairwise distances varied between 0.010 and 0.078 at the nucleotide level, suggesting that these sequences are closely related but not identical, whereas amino acid distances were lower, ranging between 0.004 and 0.024 (Table 3). Consistently, the estimated ratio of non-synonymous to synonymous substitutions was low and well below one (with the one-ratio model, dN/dS = 0.024). The sequences of the two variants found in *I. holocyclus* were extremely similar but not identical (105 differences over 6,117 nucleotide positions, but only five differences at the amino acid level). For this pair of variants, the estimated pairwise distances were 0.018 (nucleotide level) and 0.002 (amino acid level), respectively, whereas the estimated ratio of non-synonymous to synonymous substitutions was also very low (pairwise ratio, dN/dS = 0.014).

**Table 3:** Pairwise distances among complete genomes of iflavirus associated with *Ixodes ricinus* (five variants, IricIV-1 to 5, first identified in the present study) or the *I. scapularis* cell line ISE6 (ISIV). Number of base substitutions per site (Maximum composite likelihood model, 8,976 positions) followed by number of amino acid substitutions per site (Poisson correction model, 2,991 positions). Analyses were conducted with MegaX

	IricIV-1	IricIV-2	IricIV-3	IricIV-4	IricIV-5	ISIV
IricIV-1						
IricIV-2	0.059 / 0.015					
IricIV-3	0.078 / 0.020	0.064 / 0.015				
IricIV-4	0.060 / 0.016	0.010 / 0.004	0.066 / 0.016			
IricIV-5	0.076 / 0.019	0.065 / 0.015	0.028 / 0.006	0.066 / 0.017		
ISIV	0.077 / 0.024	0.049 / 0.016	0.065 / 0.019	0.050 / 0.017	0.062 / 0.019	

### Evaluation of the prevalence of iflaviruses in different populations and stages of I. ricinus

We detected the presence of the iflavirus associated with *I. ricinu*s in only two out of 12 individual field populations: those from northwestern France and Switzerland. The virus abundance was null or very low in the remaining populations (i.e., below our detection threshold). In a second data set corresponding to a lab strain, the virus was detected in all life stages and conditions, ranging from a low abundance in larvae to a maximum abundance in the egg stage (Table 4), where reads assigned to the virus represented around 0.7% of all reads sequenced.

**Table 4:**
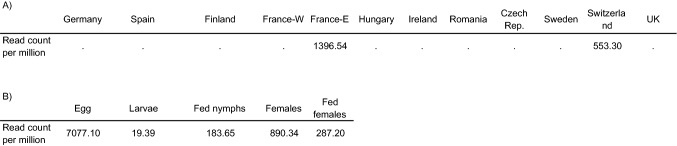
Relative abundance of an iflavirus associated with *I. ricinus*, in different field populations and life-stages. Abundance was assessed by read counts per million in different RNA-Seq libraries, comparing A) different field populations (each library was obtained from a pool of 50 nymphs), data from BioProject PRJNA662080 B) different life-stages and conditions, for a lab-reared strain, data from BioProject PRJNA595586. A dot indicates a negative library (i.e. less than the one read per million threshold), where the virus is presumed absent from the pool of individuals

## Discussion

Communities of microorganisms associated with ticks are receiving special attention in the context of increasing concern about tick-transmitted diseases [27]. In recent years, there has been a growing interest in viruses associated with ticks, and iflavirus-like viruses have emerged as some of the most common viruses in ticks [2, 5, 8, 11, 14]. Our study, based on the exploration of assembled transcriptomes, allowed us to discover nine new iflavirus genome sequences in ticks, representing five different species of tick-associated iflaviruses. We then performed a phylogenetic analysis that included these new genome sequences as well as previously published sequences. This analysis shows that the iflavirus genome sequences associated with ticks are closely grouped together and form a monophyletic clade. This suggests an ancient association between this virus subgroup and ticks, which could mean relatively infrequent host switches of the virus between major groups of arthropods. It is noteworthy, however, that two iflavirus-like genome sequences have been described recently in members of the genus *Antricola* [28], a genus of neotropical soft ticks that have a peculiar biology, being associated with hot bat caves and feeding partially on bat guano instead of having an exclusive vertebrate blood diet. These two sequences (not included in our phylogeny) grouped closely with insect iflaviruses and not with sequences associated with ticks, showing that there has been more than one infection of ticks by viruses of this family. Within ticks, the virus phylogeny and the tick phylogeny were not congruent, i.e., there was no strict co-cladogenesis (Figs. 2 and 3). One monophyletic group comprised sequences found in *I. ricinus*, *I. holocyclus*, and *I. vespertilionis*, suggesting that they form an "*Ixodes*" subclade among the iflaviruses associated with ticks. However, the virus sequence found in *I. frontalis* did not group with this ensemble and was closer to iflavirus genomes found in ticks of other genera. Also, the closer grouping of *I. holocyclus* and *I. ricinus* was inconsistent with the phylogeny of this genus [13, 29], since *I. holocyclus*, a species belonging to the Australasian subgroup of *Ixodes,* is phylogenetically distant from *I. ricinus* and *I. vespertilionis*. Although the data for iflaviruses in genera other than *Ixodes* is still relatively scarce, we finally note that the sequences associated with two species of the genus *Hyalomma* did not group together. In addition, a formal test of cophylogeny (Fig. 3) suggested several events of switching of the virus between tick species or genera over evolutionary time. A more precise picture of these dynamics will require a denser data set including more iflavirus genomes and a larger sample of tick species.

RNA viruses have relatively fast-evolving sequences, which was also suggested here by the relatively low sequence identity among genomes associated with different tick species. There was, however, a striking exception, concerning the sequence found in a cell line of *I. scapularis* (ISIV), which formed a tight cluster in the ML phylogenetic tree with five different variants identified in *I. ricinus*. In fact, these sequences were nearly identical at the amino acid level (>98% pairwise identity of protein sequences). Therefore, ISIV and the *I. ricinus* iflavirus sequences represent different strains of the same virus – a puzzling result given that the tick host species *I. scapularis* and *I. ricinus* have evolved on two different continents (eastern North America and Europe) and are separated by several million years of divergence. The geographic distance and the scarcity of animal movements (birds would be the only shared potential tick hosts) between western Europe and North America leaves little chance of a recent natural inter-species contamination through shared tick hosts. We thus propose that a contamination could explain this anomaly. Indeed, ISIV was found in the transcriptome of a cell line of *I. scapularis* (ISE6), but we failed to detect it in any other assembled transcriptomes of this species, including a data set comprising as many as 200 adult ticks from several locations [30]. In addition, another study assessed the presence of viral sequences in *I. scapularis* after surveying several pools of wild samples (a total of > 1,100 individuals, combining nymphs and adults) from different locations – New York and Connecticut, USA – but did not report any iflavirus-like sequence [5]. Of note, using the same methods, this study did identify an iflavirus genome in *Amblyomma americanum* (although it was named "dicistrovirus", we demonstrated that this sequence groups with members of the family *Iflaviridae*). By contrast, the five *I. ricinus* variants found in the present study were obtained from several independent samples of wild ticks (or from one lab strain initially derived from wild ticks), which strongly suggests that the *I. ricinus* iflavirus variants discovered in the present study correspond to strains of iflaviruses that naturally infect this species. Based on the above, we argue that the presence of the virus in the *I. scapularis* cell line is best explained by a contamination from a virus associated in the field with *I. ricinus.* Such contamination could have occurred due to the establishment or maintenance of cell lines from the two species in a same lab, a possibility that was discussed previously for other viruses and cell lines of *I. scapularis* used in a study by Alberdi et al. [31].

Much remains to be known about the evolutionary dynamics of the association between iflaviruses and tick species. For example, the effect of iflaviruses on the fitness of their tick hosts is unknown, as there are no reports, to our knowledge, of evident symptoms in ticks infected by these viruses. A second aspect to explore is the possibility that some of the tick-associated iflaviruses could have been incorporated into the tick genome, becoming endogenous viral elements (EVEs). The initial observation of a frameshift in the contig identified for *Hyalomma dromedarii* could suggest a scenario of incorporation into the host genome, followed by pseudogenization. However, further examination of the reads led us to correct the sequence, which in fact contains an intact ORF. For HflFV, RT-PCR and PCR analysis of DNA and RNA showed that the iflavirus sequence was only amplified from RNA, and thus that it was not derived from an EVE, but this test remains to be performed for other tick-associated iflaviruses. Other related points that need to be explored are the prevalence and modes of transmission of the virus in each of the tick species studied here. In one tick species, *I. ricinus*, the RNA-Seq data set allowed us to evaluate patterns of prevalence in different life stages or different field populations. For example, the high abundance of the virus in a library obtained from a pool of eggs suggests that the virus can be transmitted vertically. More experiments, based on testing of mothers and their egg mass, will be needed to determine whether this is the exclusive mode of transmission. Another data set for *I. ricinus*, based on twelve field populations (pools of nymphs) showed that the virus was only present in a minority of populations. Based on this observation, the negative results obtained for most species (i.e., all transcriptomes of 12 species of the Metastriata and three *Ornithodoros* spp*.*) should not be taken as evidence of absence of this family of viruses from the entire species. Our study supports the hypothesis of an ancient association and relatively widespread presence of iflavirus genomes in tick species. We suggest that a more systematic use of RNA-Seq, based on large pools of wild individuals, would maximize the chance of detecting sequences of tick-associated viruses, even in cases of low prevalence [32]. More generally, this would enhance progress of the description of the virome associated with ticks, comprising viruses that are pathogenic for their vertebrate hosts, including humans [33].

## Declarations

### Availability of data and material

Genome sequences of iflaviruses have been submitted to the GenBank database.
